# Intrahepatic cholangiocarcinoma: The role of liver transplantation, adjunctive treatments, and prognostic biomarkers

**DOI:** 10.3389/fonc.2022.996710

**Published:** 2022-11-21

**Authors:** Ashton A. Connor, Sudha Kodali, Maen Abdelrahim, Milind M. Javle, Elizabeth W. Brombosz, R. Mark Ghobrial

**Affiliations:** ^1^ Sherrie and Alan Conover Center for Liver Disease and Transplantation, JC Walter Jr Transplant Center, Houston Methodist Hospital, Houston, TX, United States; ^2^ Department of Surgery, Houston Methodist Hospital, Houston, TX, United States; ^3^ Department of Medicine, Weill Cornell Medical College, New York, NY, United States; ^4^ Section of Gastrointestinal Oncology, Department of Medical Oncology, Houston Methodist Cancer Center, Houston, TX, United States; ^5^ Cockrell Center Phase 1 Unit, Cockrell Center for Advanced Therapeutics, Houston Methodist Hospital, Houston, TX, United States; ^6^ Department of Gastrointestinal Medical Oncology, MD Anderson Cancer Center, Houston, TX, United States

**Keywords:** liver transplant (LT), intrahepatic cholangiocarcinoma, transplant oncology, personalized & precision medicine (PPM), next generation (deep) sequencing (NGS)

## Abstract

Intrahepatic cholangiocarcinoma (iCCA) is a primary epithelial cell malignancy of the liver with rising incidence rate globally. Its insidious presentation, heterogeneous and aggressive biology, and recalcitrance to current therapies results in unacceptably high morbidity and mortality. This has spurred research efforts in the last decade to better characterize it molecularly with translation to improved diagnostic tools and treatments. Much of this has been driven by patient advocacy. This has renewed interest in orthotopic liver transplantation (LT) with adjunctive therapies for iCCA, which was historically disparaged due to poor recipient outcomes and donor organ scarcity. However, the optimal use of LT as a treatment for iCCA care remains unclear. Here, we review the epidemiology of iCCA, the history of LT as a treatment modality, alternative approaches to iCCA local control, the evidence for peri-operative systemic therapies, and the potential roles of biomarkers and targeted agents. In doing so, we hope to prioritize areas for continued research and identify areas where multidisciplinary care can improve outcomes.

## Introduction

### Epidemiology of iCCA

**Figure 1:**
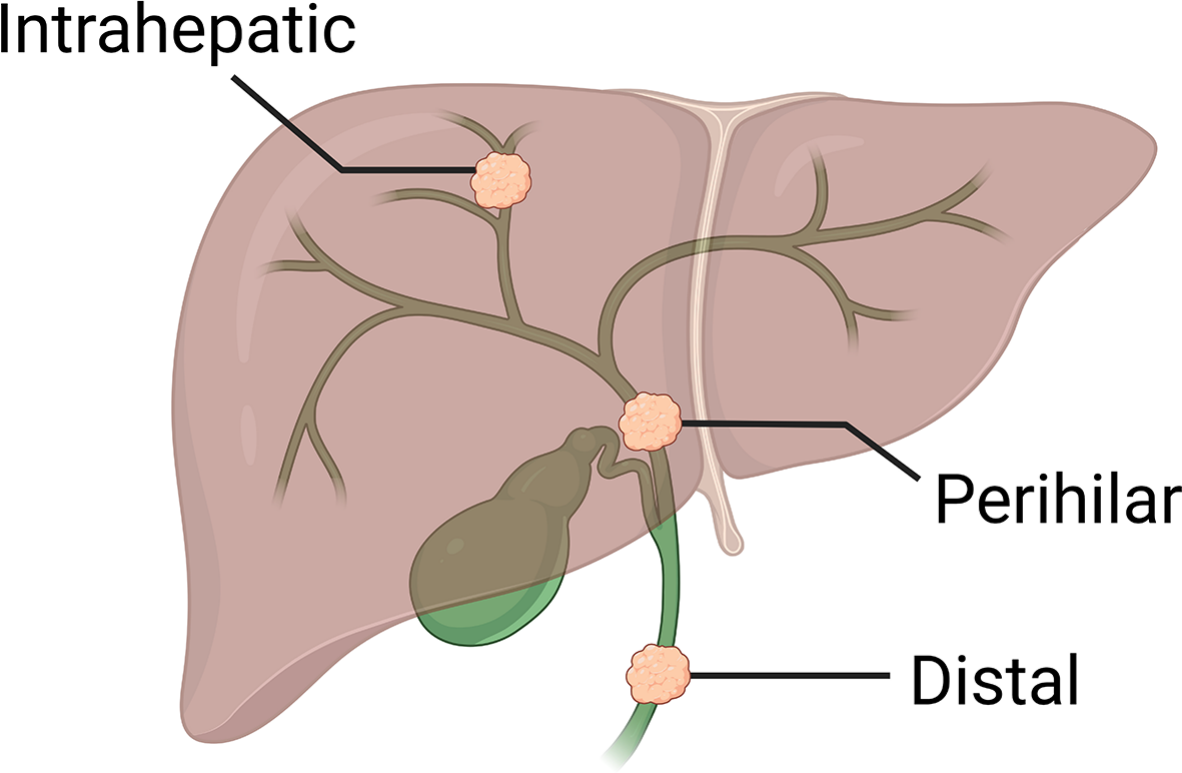
Three anatomical subtypes of cholangiocarcinoma.

Cholangiocarcinoma (CCA) is an adenocarcinoma arising from anywhere along the biliary system. CCAs are distinguishable epidemiologically, anatomically, and molecularly into three subtypes: intrahepatic (iCCA; 10-20% of all CCAs), perihilar (50-60%), and distal (20-30%; **Figure 1**). Each subtype is therefore managed differently in the surgical setting, although systemic approaches are non-selective.

iCCA is a primary liver cancer that arises from second-order bile ducts or higher within the liver parenchyma. Globally, iCCA accounts for 10-15% of primary liver cancer cases, with more than 90,000 new cases and 83,000 deaths occurring in 2020 (1). Incidence and age-standardized mortality rates for iCCA have been increasing globally since the year 2000 (2). Within the United States (US), an alarming 5.9% annual increase in the incidence of iCCA was reported between 2003 and 2009, only some of which is attributed to detection bias from enhanced diagnostic modalities and reclassification (3). Also in the US, the largest increases in mortality were seen in African-Americans, followed by Asian-Americans, then Caucasians (4). Unfortunately, the true incidence of iCCA is obscured by confusion of intrahepatic and perihilar CCA in national databases, in part owing to a single International Classification of Diseases (ICD) code for both entities (5).

Many patients have concurrent liver dysfunction, including chronic liver parasitic trematode infection (liver flukes in endemic areas such as Southeast Asia), choledocholithiasis, congenital choledochal cysts, primary sclerosing cholangitis, chronic hepatitis B and C infections, smoking and alcoholic and non-alcoholic fatty liver disease (6). The rising incidence rate of the latter contributes to the increased burden of iCCA in Western countries (7), though most iCCAs remain idiopathic. Many of these risk factors involve chronic biliary inflammation and stasis, which may explain the protective effect of aspirin and statin medications in case-control studies (8–10). Based on a retrospective United Network of Organ Sharing (UNOS) database review, compared with hepatocellular carcinoma (HCC), the more common primary hepatic malignancy, patients with iCCA had a lower mean age at treatment (49.9 years, standard deviation 11.9), were less often male (66%), and more often of European ancestry (89%) (11).

Most iCCA patients will succumb within two years of diagnosis (12). Death is most often related to intrahepatic local progression and comorbidity associated with biliary obstructions rather than distant dissemination. Fortunately, iCCA management is now being informed by translation of basic science, advances in multidisciplinary care, and strong patient advocacy.

### Growth patterns

Three macroscopic iCCA growth patterns are observed: mass-forming (most common), periductal infiltrating, and intraductal papillary. The patterns are prognostic, with “intraductal” papillary having the most favorable outcomes (13).

Histologically, iCCA can also be classified as either small bile duct or mucin-producing large bile duct types (14). Duct types are associated with molecular carcinogenesis, with large-duct iCCA showing a high frequency of canonical adenocarcinoma genetic alterations, such as in *KRAS* and *TP53*, while *IDH1/2* and *FGFR2* activations are typically seen in small-duct type tumors (15). These two histologic types may have different cancer stem cell origins (16).

### Peri-tumoral stroma

The tumor microenvironment (TME) associated with iCCA is a highly desmoplastic network of extracellular collagen- and protein-rich matrix, myofibroblasts, macrophages, and other immune cells (17). This stroma promotes iCCA progression and therapeutic resistance. Myofibroblast depletion limits tumor growth in murine models (18), and CD163(+) macrophage counts are associated with poor survival (19). Prognostic features of the iCCA TME can be identified by immunohistochemistry, such as high expression of α-smooth muscle actin and periostin (20). Targeting the TME may be a promising therapeutic approach in the future.

### Patient-centered outcomes

Patients with iCCA proactively seek personalized treatments, especially as there are few centers offering precision oncology-based treatment. Access to treatment is a significant barrier faced by patients, clinicians, and researchers. Patients must travel to receive care and/or to participate in clinical trials. In turn, these hardships cause other issues, such as isolation and financial burden (21). Disseminating best practices to medium-sized and even small centers may alleviate unnecessary patient suffering and advance the field.

### iCCA conventional management

#### Screening, diagnosis, and staging

Patients with iCCA are typically asymptomatic until advanced stages, when they may develop pain or jaundice from local growth. As such, diagnosis is incidental in approximately 25-33% of patients. There are no accepted screening protocols for non-fluke CCA, despite other high-risk populations, such as patients with primary sclerosing cholangitis. Even amongst the latter, the cumulative incidence at 20 years is less than 25%, and screening for CCA is challenged by biliary inflammation, possible requirement of invasive procedures, and absence of benefit in prospective series (22).

Diagnosis of iCCA by non-invasive testing is challenging. It can be difficult to distinguish CCA from other primary liver malignancies without tumor sampling. The only commonly-employed serum biomarker is carbohydrate antigen 19-9 (CA19-9), which lacks both sensitivity and specificity (23). Imaging modalities include ultrasound, CT, and MRI with or without contrast enhancement. In patients with cirrhosis who undergo image-based screening for HCC, iCCA may be recognized early, though distinguishing these two primary hepatic malignancies radiologically can be nuanced in that setting (24, 25). Patients undergoing liver resection and/or transplantation for presumed HCC may be diagnosed with iCCA on final pathology (**Table 1**). Yet, the evidence for neoadjuvant systemic therapy for iCCA (see below) implies that the correct diagnosis should be made pre-operatively.

**Table 1:** Case series on liver transplantation for incidentally discovered intrahepatic cholangiocarcinoma.

Study	Year	Study design	n	1-year OS (%)	3-year OS (%)	5-year OS (%)	DFS	Neoadjuvant Treatment	Adjuvant Treatment
Yokoyama et al. (26)	1990	Retrospective	2	50	0	–	–	none	none
Ghali et al. (27)	2005	Retrospective Multicentre	10	–	30	–	–	none	none
Sotiropoulos et al. (28)	2008	Retrospective	10	70	50	33	–	none	none
Vallin et al. (29)	2013	Retrospective Multicentre	10	80	60	24	–	none	none
Sapisochin et al. (30)	2014	Retrospective Multicentre	single ≤2 cm: 8	100	73	73	71% at 5 years	locoregional therapy	none
			multiple or >2 cm: 21	71	43	34			
Takahashi et al. (31)	2016	Retrospective	13	–	–	–	42% at 3 years	locoregional therapy	none
Sapisochin et al. (32)	2016	Retrospective Multicentre	single ≤2 cm: 15	93	84	65	82% at 5 years	none	none
			multiple or >2 cm: 33	79	50	45	39% at 5 years		
De Martin et al. (33)	2020	Retrospective Multicentre	24	–	–	69	75% at 5 years	none	none
Krasnodębski et al. (34)	2020	Retrospective	6	75	37.5	25	28.6% at 5 years	none	none

DFS, disease-free survival; OS, overall survival.

Staging for iCCA can include the radiology modalities above. ^18^F-Fluorodeoxyglucose (^18^F-FDG) positron-emission tomography, a test with high sensitivity and specificity for lymph node and distant metastases, is often utilized for staging (35). The low specificity for primary lesions implies that biopsy is still required for diagnosis, particularly before systemic treatment (36).

#### Local resection

**Figure 2:**
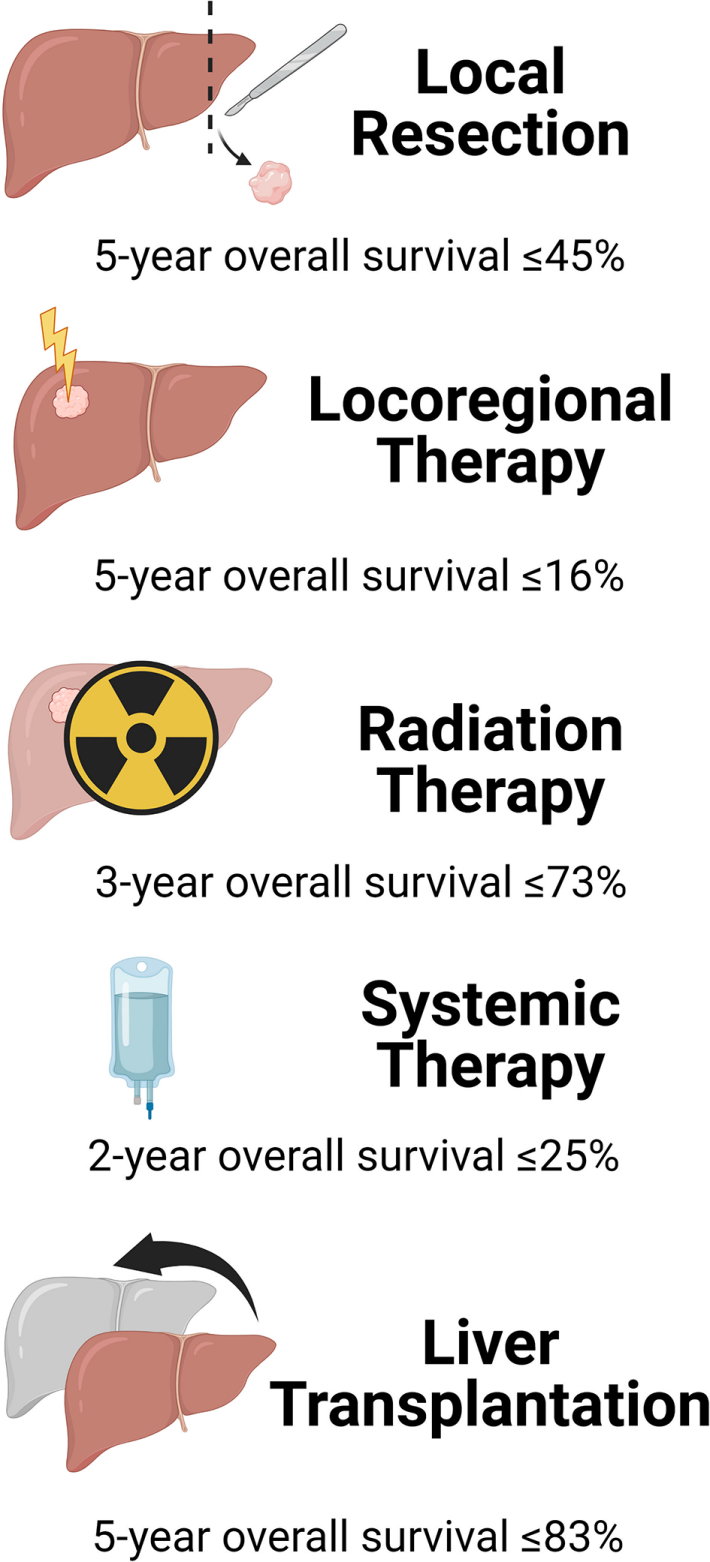
Outcomes of intrahepatic cholangiocarcinoma by treatment modalities.

According to a retrospective review of the National Cancer Database from 2004 to 2015 (37), 81% of patients who had invasive procedures for iCCA underwent local resection (LR), 11% locoregional ablation, and 8% liver transplantation (LT). These therapies had different outcomes (**Figure 2**). Margin-negative (R0) surgical liver resection (LR) is deemed the only potentially curative treatment for iCCA by expert consensus guidelines (38). However, many patients are not amenable to LR due to tumor size and/or number, or underlying cirrhosis.

Most patients with iCCA present with locally advanced tumors too large for LR. Analysis of the SEER database between 1983 and 2010 confirmed that only 15% of all patients with iCCA underwent LR (39). In most cases of LR, patients with iCCA required a major hepatectomy for complete tumor extirpation. Median overall survival (OS) after LR for iCCA is reported to be as little as 40 months, with 5-year OS ranging from 15% to 45% (12, 32, 40).

Tumor recurrence is seen in 50-70% of patients at a median time of 26 months from LR (41). The remnant liver is the most frequent site of recurrence following LR for iCCA, with exclusive intrahepatic recurrence in 60% of cases (42). Repeat LR may be attempted depending on the anatomy of the remnant liver and the recurrent tumor. Well-selected patients amenable to repeat LR can expect satisfactory survival. A recent meta-analysis of repeat LR reported 1-, 3-, and 5-year OS rates of 87%, 58%, and 39%, respectively (43).

Multifocal iCCA is seen in nearly 50% of patients at initial presentation and is a poor prognostic factor (44), especially if there are 3 or more lesions (45). Multifocal iCCA poses a greatly increased risk of recurrence and death after LR, which does not seem to improve OS and recurrence-free survival (RFS) compared to locoregional therapies in this setting (46).

American Joint Committee on Cancer (AJCC), National Comprehensive Cancer Network (NCCN) and International Liver Cancer Association (ILCA) guidelines recommend lymphadenectomy, harvesting at least 6 lymph nodes for staging of iCCA (47). Nodal metastases are strongly correlated with outcomes and are present in up to 45% of patients (12). In one series, patients who had 3 or more lymph nodes resected had better survival than those with only 1-2 nodes removed (48). In another series, LR for node-positive disease did not confer a survival advantage over chemotherapy alone, demonstrating the poor prognosis of iCCA with nodal metastasis (49).

#### Locoregional therapy

Locoregional therapy (LRT) is used when tumors are unresectable, either for patient or tumor factors. In a multi-center retrospective analysis, intra-arterial embolization therapy (IAET) for advanced iCCA achieved complete or partial response in 25% of patients and disease stability in 61% (50). Median survival in this cohort was 13.2 months, making these worthwhile palliative therapies (50). OS at 1, 3, and 5 years was 54.0%, 22.2%, and 16.2%, respectively (50). IAET can also downstage tumors for resection (51), and some experts feel that response to locoregional therapies is a reasonable selection tool for patients with iCCA who may benefit from surgery, regardless of initial presentation.

LRT is an uncommon neoadjuvant for LT candidates diagnosed iCCA (**Tables 1**, **2**). For patients misdiagnosed with HCC prior to LT, LRT may be used, with iCCA incidentally discovered post-LT (64). In one study on such patients, only 1 of 13 who received neoadjuvant LRT had ≥60% tumor necrosis at LT (30), 2 had 30%-60% necrosis, and the remainder had <30% or none. In another study, 5 of 13 patients with incidental iCCA underwent either IAET or radiofrequency ablation (RFA) (31) but had similar outcomes as the patients who did not. These results suggest that LRTs developed to treat HCC are ineffective neoadjuvant treatments of iCCA.

**Table 2:** Case series on liver transplantation for pre-operatively discovered intrahepatic cholangiocarcinoma.

Study	Year	Study design	n	1-year OS (%)	3-year OS (%)	5-year OS (%)	DFS	Neoadjuvant Treatment	Adjuvant Treatment
O'Grady et al. (52)	1988	Retrospective	13	38	10	10	–	none	none
Goldstein et al. (53)	1993	Retrospective	17	53	–	–	40% at 1 year	none	chemotherapy and radiation
Pichlmayr et al. (54)	1997	Retrospective	24	19	5	0	–	none	none
Meyer et al. (55)	2000	Retrospective Multicentre	207	72	48	23	84% at 2 years	none	some
Shimoda et al. (56)	2001	Retrospective	16	62	39	–	35% at 5 years	none	none
Robles et al. (57)	2004	Retrospective Multicentre	23	77	65	42	35% at 2 years	none	none
Hu et al. (58)	2011	Retrospective	20	84	33	22	19% at 5 years	chemotherapy	none
Hong et al. (59)	2011	Retrospective	25	–	38	32	33% at 5 years	chemotherapy	chemotherapy
Facciuto et al. (60)	2015	Retrospective	32	71	–	57	44% at 5 years	none	none
Vilchez et al. (11)	2016	Retrospective Multicentre	440	79	58	47	–	none	none
Lunsford et al. (61)	2018	Prospective single-arm	6	100	83.3	83.3	50% at 5 years	chemotherapy	chemotherapy
Ito et al. (62)	2022	Retrospective	31	80	63	49	42% at 5 years	chemotherapy *some* locoregional *some*	chemotherapy *some*
McMillan et al. (63)	2022	Prospective single-arm	18	100	71	57	52% at 3 years	chemotherapy	chemotherapy

DFS, disease-free survival; OS, overall survival.

#### Radiation therapy

Several retrospective and small prospective studies have examined the use of radiation therapy for iCCA. High-dose ablative radiotherapy (mean biologic equivalent dose of >80.5 Gy) can be an effective treatment in patients with inoperable iCCA, with a 3-year OS rate of 73% (65). Proton therapy also shows potential. It has been used to successfully control growth of unresectable iCCA tumors, with 1- and 3-year OS rates of 82% and 38%, respectively (66). For example, a single-arm, phase II, multi-center study of hypofractionated proton therapy on 37 iCCA patients demonstrated 2-year local disease control rate of 94.1% (67). In addition, a 2020 study from the team at Massachusetts General Hospital showed that hypofractionated proton radiotherapy improves OS and offers 84% local control after 2 years (68). However, proton therapy is not widely available, and therefore its utility may be limited for many iCCA patients. Optimal selection criteria for radiation for local control remain unclear, especially in the context of LRT and systemic therapies (69).

To our knowledge, only one study has reported on radiation therapy neoadjuvantly in LT for iCCA (20). This study reported 100% OS at 5 years post-LT in 4 iCCA patients when radiation was given in combination with chemotherapy neoadjuvantly, but not if used as monotherapy (20). One 1993 study reported on adjuvant radiation therapy for LT recipients (53). These patients received radiation beginning 8 weeks after LT without morbidity, focusing on areas of likely regional recurrence including lymph nodes and porta hepatis. Unfortunately, this did not improve survival. Given its promise as stand-alone therapy, future research should investigate radiation modalities as adjuncts to LT for iCCA patients.

#### Systemic therapy

Chemotherapy is the most common adjunctive therapy to LT for iCCA (**Table 2**). Yet, randomized trials supporting neo- and adjuvant chemotherapy in iCCA are lacking. Non-randomized studies have shown neoadjuvant therapy can achieve stability or reduction of tumor size and/or number. For example, one series showed that neoadjuvant gemcitabine downstaged approximately 40% of previously unresectable patients for LR, achieving 5-year OS of 45% (70).

In the adjuvant setting, the BILCAP phase 3 study did not meet its primary endpoint of improving overall survival post-LR, though sensitivity and per protocol analyses did suggest adjuvant oral capecitabine was beneficial (71). Similarly, both the PRODIGE-12 phase 3 trial and the BCAT phase 3 trial did not demonstrate a benefit from gemcitabine-based therapy post-LR (72, 73). A retrospective review of the National Cancer Database from 2004 to 2015 showed that only 42% of iCCA patients were receiving adjuvant chemotherapy (37), although practice guidelines do recommend 6 months of oral capecitabine (74).

In the palliative setting, where patients are ineligible for curative treatment, chemotherapy alone is an option, often with cisplatin and gemcitabine as first-line choices. A *post-hoc* analysis of 66 iCCA patients in the ABC-01, -02, and -03 trials treated with cisplatin-gemcitabine chemotherapy demonstrated median progression-free survival and OS of 8.4 months and 15.4 months, respectively (75). The ABC-06 trial showed increased 6- and 12-month overall survival with use of FOLFOX as second-line therapy (76).

Also in the palliative setting, the recent TOPAZ-1 trials have also shown the promising results of gemcitabine and cisplatin combined with immunotherapy for biliary tract cancer. In combination with gemcitabine-cisplatin, the phase 2 study showed durvalumab with or without tremelimumab, with 66% of patients responding to treatment (77). Preliminary results of the phase 3 trial showed that the combination of gemcitabine, cisplatin, and durvalumab significantly improved OS and progression-free survival (78). Combined systemic therapies show great promise in treating unresectable biliary tract cancers, including iCCA.

Systemic therapy is the most common adjunct to LT in iCCA patients (**Table 2**). It is also used for recurrent iCCA post-LT (**Table 2**). Response to neoadjuvant chemotherapy can inform selection of iCCA LT candidates, as described by our institution to identify favorable tumor biology (63). All patients in this study received systemic therapy, generally gemcitabine and cisplatin. Disease response for more than 6 months was required prior to LT, resulting in 100% survival at one year after LT, implying its importance as a patient selection criterion.

### Liver transplantation for iCCA

LT involves orthotopic replacement of a patient’s diseased liver with a donor healthy liver. It offers a chance of cure for well-selected patients with either primary or metastatic liver tumors that have limited alternative treatment options. LT can achieve margin-negative liver tumor extirpation, including of pre-operatively occult lesions. For treatment of iCCA, LT offers advantages over LR, including an improved likelihood of achieving negative oncologic margins, eliminating intrahepatic micro-metastases, and resolving any underlying liver disease.

#### Historical outcomes

Initial series of LT for iCCA reported outcomes that were lower than those observed for other LT indications, and approximately equivalent to LR outcomes (see **Tables 1** and **2**) (11, 26–29, 52–58, 60, 79–81). After 2014, several retrospective cohorts identified improved results with use of LT in highly selected iCCA patients, chosen because they were thought to have favorable tumor biology and response to adjunctive therapy trials (see **Tables 1** and **2**).

Interest in LT for iCCA was also re-invigorated by the improved outcomes achieved from LT for HCC and perihilar CCA. Once considered a contraindication to LT, early studies described the successful treatment of small HCC lesions with LT (82, 83), leading to selection criteria based on tumor size and number (84). These criteria were gradually expanded to include patients with a greater HCC tumor burden (85), followed by the incorporation of biomarkers (86) and adjunctive therapies (87) to expand the pool of eligible patients.

For perihilar CCA, registry-based LT outcomes were quite poor (55), even for small, incidentally identified tumors on explant (27). However, introduction of neoadjuvant chemotherapy and radiation therapy for systemic and local control prior to transplantation (88–91) resulted in a RFS of 65% at 5 years. This implies that the biological activity of CCA, including response to adjunctive therapies, is a better marker of post-LT for optimal outcomes than size-based criteria alone.

#### Recent outcomes

Encouraged by outcomes in select HCC and pCCA patients, LT in iCCA patients was re-evaluated (**Tables 1** and **2**). In 2014, a Spanish multi-center retrospective study reported that 5-year OS following LT in patients with cirrhosis and small (<2 cm) incidental iCCA was 65% (**Table 1**) (80). In the follow-up multi-national retrospective cohort derived from 17 transplant centers, 1-, 3-, and 5-year OS were 93%, 84%, and 65%, respectively, for 48 LT patients with cirrhosis and small (<2 cm) incidental iCCA (32). LT recipients also had low tumor recurrence. These outcomes approach those achieved for patients transplanted for other malignancies, such as HCC, and are superior to those achieved with LR.

Further studies investigated the utility of LT for unresectable iCCA. A group from UCLA first reported good OS in 38 patients diagnosed with unresectable iCCA prior to LT (59). Most tumors (95%) were locally advanced. The same group subsequently found that neoadjuvant therapy, tumor multifocality, perineural invasion, and infiltrative growth were all important prognostic factors (92). In 2022, UCLA reported updated outcomes of LT for iCCA, with 1-, 3-, and 5-year OS rates of 80%, 63%, and 49%, respectively (62).

Some studies report LT outcomes for both iCCA and perihilar CCA. For example, a retrospective UNOS database review reported that both CCA patients had 1-, 3-, and 5-year post-LT OS rates of 79%, 58%, and 47%, respectively (11). De Martin and colleagues found that LT recipients who had iCCA alone or combined with HCC had OS of 90%, 76%, and 67% at 1-, 3-, and 5- years post-LT, respectively (33). These results agree with reports of post-LT outcomes for iCCA alone (**Tables 1**, **2**).

These and other retrospective studies are summarized in one meta-analysis (93). According to the analysis, factors associated with tumor recurrence were microvascular invasion, poor tumor differentiation, tumor size, and number. Because many of these studies included tumors that were identified incidentally in explanted, cirrhotic livers or pre-dated the modern era, patients had neither received neoadjuvant systemic nor locoregional therapies.

The effects of adjunctive therapies were explored in two 2011 series from UCLA. The first showed that CCA patient outcomes were determined by aspects of tumor biology rather than size. Predictive factors included multifocality, infiltrative growth pattern, perineural and lymphovascular invasion, history of primary sclerosing cholangitis, and the use of neoadjuvant and adjuvant therapy (92). The second paper reported improved 5-year OS for iCCA patients undergoing neo- and adjuvant systemic therapy with LT compared to LR in the absence of background liver dysfunction, independent of tumor size (59).

#### Patient selection

Informed both by these preceding studies and by iCCA molecular biology, the center-approved clinical practice guideline at Houston Methodist selects iCCA patients for LT using the following criteria: AJCC stage I or II diagnosis with imaging and/or biopsy that is unresectable, no macrovascular invasion, disease stability or reduction for at least 6 months following neoadjuvant systemic therapy with or without LRT, negative laparotomy and lymphadenectomy prior to transplant, and adjuvant systemic therapy following LT. Unlike most early studies, these patients are diagnosed with iCCA prior to transplant (**Table 2**).

The team at Houston Methodist Hospital has published two prospective case-series of patients with unresectable iCCA managed with LT and neoadjuvant chemotherapy (61, 63). Response to neoadjuvant treatment served as a surrogate for favorable disease biology, even in cases of locally advanced iCCA. In both series, roughly one third of patients referred and one half of patients listed ultimately underwent LT. They achieved excellent OS at 1-, 3-, and 5-years of 100%, 83%, and 83%, respectively, in the first study (61), and 100%, 71%, 57%, respectively, in the second study (63). The iCCA recurred in 39% of patients at a median time of 11 months post-LT. Recurrences were treated with further systemic therapy and surgery. Patients who were listed but not transplanted had an abrupt decline in survival after 1 year, and none were observed to be alive for more than 2 years, consistent with prior reports of iCCA patients managed only with systemic therapy (75). Indeed, these results exceed those previously reported for either LR, LT, or chemotherapy alone.

**Figure 3:**
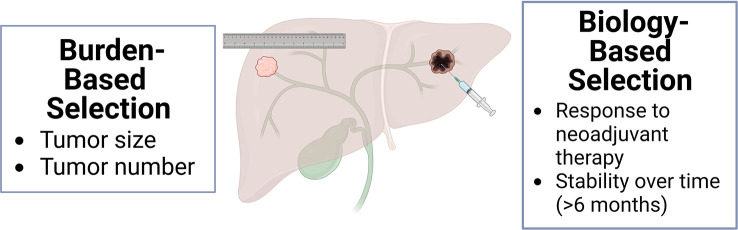
Selection criteria for liver transplantation for iCCA, burden-based and biology-based.

These studies demonstrate that for well-selected iCCA patients with liver-only disease, LT is a curative treatment option that may achieve superior outcomes to LR or systemic therapy alone. Two main published strategies of patient selection are burden-based and biology-based (**Figure 3**). Initial successful studies advocated for iCCA size and number restrictions (32). Subsequent series have shown good outcomes based on neoadjuvant response, independent of size (61–63).

It is also important to consider whether LT candidates would have similar survival undergoing other iCCA treatments. Unfortunately, retrospective comparisons of treatment modalities from CCA registries have yielded limited insights. A recent review of the National Cancer Database from 2010 to 2016 used a 1:1 propensity score match to compare iCCA patient outcomes with LR and LT, finding no significant differences. Unfortunately, this study lacked granularity on patient selection measures, tumor biology, and responses to adjunctive therapies (94).

We hope for additional prospective series that incorporate more aspects of tumor biology to come to a consensus regarding LT selection criteria for iCCA.The relative rarity of iCCA, its inherent aggressiveness, and the challenges of transplant surgery prohibit randomization for trials.

In addition, the standardized treatment guidelines for neoadjuvant and bridging therapy are still unclear for iCCA patients who are listed for LT. Recent data from our group showed that CCA patients experience excellent survival when treated with neoadjuvant gemcitabine and cisplatin without radiation (95). In this prospective study, ten patients received a combination therapy of gemcitabine and cisplatin as neoadjuvant while awaiting LT, with a median follow up of 851 days. OS was 100% (95% CI: 100-100%) at 1- and 2-years post-LT, and 75% (95% CI: 13-96%) at 3- and 5-years post-LT. The UCLA group showed similar outcomes when neoadjuvant adjunctive therapies were used (62).

Post-LT outcomes may also vary by transplant center. Center volume was associated with post-LT OS and graft survival in a database study of all CCA (96), whereas a multi-center study of LT for perihilar CCA found equivalent outcomes at high and low volume centers (91). More studies are needed to investigate center-specific effects and how lessons from high-volume centers might be translated to lower-volume centers.

## Biomarkers

CCAs are very heterogeneous tumors. Variations in molecular carcinogenesis, tumor microenvironment, histology, and growth patterns have been shown to be prognostic and predictive, though their role in informing LT requires much further investigation. We summarize the translational findings of biomarkers on outcomes here in the hope of stimulating discussion of their roles in transplantation for iCCA.

CCA has been well characterized molecularly, and mutations vary by anatomic location and by etiology (97). Like other adenocarcinomas, iCCA has a high frequency of mutations in tumor protein p53 (*TP53*), Kirsten rat sarcoma viral oncogene homolog (*KRAS*), and mothers against decapentaplegic homolog 4 (*SMAD4*), and these may be associated with worse prognosis (97). These tumors also frequently bear alterations of fibroblast growth factor receptor 2 (*FGFR2*), isocitrate dehydrogenase 1 (*IDH1*), isocitrate dehydrogenase 2 (*IDH2*), RB Transcriptional Corepressor 1 (*RB1*), Erb-B2 receptor tyrosine kinase 2 (*ERBB2*), and breast cancer associated protein 1 (*BAP1*) (98). It has been estimated that as many as 70% of patients with iCCA have potentially targetable mutations (99).

*FGFR2* translocations preferentially occur in iCCA at a frequency of 15%, relative to other adenocarcinomas, creating constitutively active fusions to many different gene partners (98, 100). These are associated with more indolent disease course (101), and they predict response to FGFR inhibitors (99, 102, 103). Gain-of-function mutations at hotspot locations in *IDH1^R132^* and *IDH2^R172^* also occur in approximately 15% of iCCAs, causing an accumulation of the onco-metabolite 2-hydroxyglutarate (104). Expression of these genes also predicts response to targeted inhibitors (105). Activating *BRAF* mutations are found in up to 5% of iCCAs, and early results of a phase 2 trial using the targeted combination of dabrafenib and trametinib in these patients are promising (106).

Approximately 6% of CCAs are hypermutated, with a median number of 641 non-silent mutations per exome (98). These tumors have characteristic elevation of anti-tumor immunity marker expression. Approximately one third of these are mismatch repair deficient. There is evidence from phase 2 trials that a subset of CCAs (approximately 10%) respond to immune checkpoint inhibitors, though biomarkers to identify these responders have not yet been identified (107–109). Remarkably, germline predisposition to CCA is poorly described, and there are no published genome-wide association studies.

In the prospective series of LT recipients at Houston Methodist, the iCCA tumors in the liver explant undergo molecular profiling. The most frequently altered genes were *FGFR2* and DNA damage pathways genes (63). The few patients whose tumors bore *KRAS* and *BAP1* mutations developed recurrent disease, supporting the association between these mutations and aggressive tumor biology (110).

Circulating tumor DNA (ctDNA) may be a useful tool for monitoring genomic mutations. In a small series, ctDNA was detected in iCCA patients by either targeted sequencing (111, 112) or multiplex digital PCR (113). The ctDNA results were highly concordant with solid tumor mutations, and with variant allele frequencies correlated to tumor burden. Thus, ctDNA currently shows promise as a biomarker for iCCA presence and biology.

Research has begun to show how and when these genomic aberrations might best inform clinical practice. There are several kinase inhibitors that are now FDA approved as second-line treatments for CCA. Pemigatinib (103) and infigratinib (102, 114) have been shown to successfully treat patients with *FGFR* fusions or rearrangements, with PFS of 6.9 and 5.8 months, respectively. Pemigatinib was the first targeted therapy approved by the FDA as a palliative treatment for CCA, and acts on *FGFR2* fusions. Infigratinib is an *FGFR1-3* kinase inhibitor also approved in the palliative setting. The ClarIDHy trials of ivosidenib have shown improved progression-free survival (105) and OS (115) in CCA patients with *IDH1* mutations, and it has also been approved as palliative therapy. Two therapies have recent breakthrough designations by the FDA for biliary tract cancers, namely zanidatamab, an anti-HER2 antibody, and futibatinib, another FGFR2 antagonist, both in the palliative setting (116, 117).

The roles that these biomarkers may serve as biology-based selection criteria or in guiding adjunctive therapies for LT in iCCA remains to be identified.

## Discussion and outlook

The last decade has seen tremendous progress in our understanding of iCCA carcinogenesis, molecular profiling, and responses to existing treatments. Despite these advances, there is still much progress to be made. Preclinical models are lacking. The majority of cases remain idiopathic. Evidence for prevention and screening is elusive. The various treatment options, including LR, LRT, radiation therapy, systemic, and targeted therapies, lack integration with LT. Improved guidelines for LT recipient selection are also needed, with current evidence for either tumor burden- or biology-based criteria. Our center has endorsed inclusion criteria of disease stability or response to neoadjuvant therapy for at least six months. The ideal timing, method of acquisition, and translation of molecular data is unclear. Thus, considerably more remains to be learned to reduce the morbidity and mortality of this recalcitrant malignancy.

## Author contributions

AC wrote the initial manuscript. SK, MA, MJ, EB, RG contributed to the content and made revisions. All authors contributed to the article and approved the submitted version.

## Acknowledgments

The figures in this manuscript were created using BioRender software.

## Conflict of interest

The authors declare that the research was conducted in the absence of any commercial or financial relationships that could be construed as a potential conflict of interest.

## Publisher’s note

All claims expressed in this article are solely those of the authors and do not necessarily represent those of their affiliated organizations, or those of the publisher, the editors and the reviewers. Any product that may be evaluated in this article, or claim that may be made by its manufacturer, is not guaranteed or endorsed by the publisher.
